# Phosphatidylinositol 3-Kinase/AKT Pathway Inhibition by Doxazosin Promotes Glioblastoma Cells Death, Upregulation of p53 and Triggers Low Neurotoxicity

**DOI:** 10.1371/journal.pone.0154612

**Published:** 2016-04-28

**Authors:** Mariana Maier Gaelzer, Bárbara Paranhos Coelho, Alice Hoffmann de Quadros, Juliana Bender Hoppe, Silvia Resende Terra, Maria Cristina Barea Guerra, Vanina Usach, Fátima Costa Rodrigues Guma, Carlos Alberto Saraiva Gonçalves, Patrícia Setton-Avruj, Ana Maria Oliveira Battastini, Christianne Gazzana Salbego

**Affiliations:** 1 Programa de Pós-Graduação em Ciências Biológicas: Bioquímica, Instituto de Ciências Básicas da Saúde, Universidade Federal do Rio Grande do Sul (UFRGS), Porto Alegre, RS, Brasil; 2 Departamento de Bioquímica, Instituto de Ciências Básicas da Saúde, Universidade Federal do Rio Grande do Sul (UFRGS), Porto Alegre, RS, Brasil; 3 Departamento de Química Biológica, Facultad de Farmácia y Bioquímica, Universidad de Buenos Aires (UBA), Ciudad Autónoma de Buenos Aires, Argentina; Swedish Neuroscience Institute, UNITED STATES

## Abstract

Glioblastoma is the most frequent and malignant brain tumor. Treatment includes chemotherapy with temozolomide concomitant with surgical resection and/or irradiation. However, a number of cases are resistant to temozolomide, as well as the human glioblastoma cell line U138-MG. We investigated doxazosin’s (an antihypertensive drug) activity against glioblastoma cells (C6 and U138-MG) and its neurotoxicity on primary astrocytes and organoptypic hippocampal cultures. For this study, the following methods were used: citotoxicity assays, flow cytometry, western-blotting and confocal microscopy. We showed that doxazosin induces cell death on C6 and U138-MG cells. We observed that doxazosin’s effects on the PI3K/Akt pathway were similar as LY294002 (PI3K specific inhibitor). In glioblastoma cells treated with doxasozin, Akt levels were greatly reduced. Upon examination of activities of proteins downstream of Akt we observed upregulation of GSK-3β and p53. This led to cell proliferation inhibition, cell death induction via caspase-3 activation and cell cycle arrest at G0/G1 phase in glioblastoma cells. We used in this study Lapatinib, a tyrosine kinase inhibitor, as a comparison with doxazosin because they present similar chemical structure. We also tested the neurocitotoxicity of doxazosin in primary astrocytes and organotypic cultures and observed that doxazosin induced cell death on a small percentage of non-tumor cells. Aggressiveness of glioblastoma tumors and dismal prognosis require development of new treatment agents. This includes less toxic drugs, more selective towards tumor cells, causing less damage to the patient. Therefore, our results confirm the potential of doxazosin as an attractive therapeutic antiglioma agent.

## Introduction

Gliomas are malignant primary brain tumors with no effective cure. Diffuse high grade gliomas (glioblastoma) patients have a short life expectancy despite aggressive therapeutic approaches based on surgical resection followed by adjuvant radiotherapy and concomitant chemotherapy [1].

Molecular mechanisms of glioblastoma multiform (GBM) resistance to therapy involve the PI3K/Akt pathway—which regulates cell proliferation, cell cycle, survival, apoptosis, chemotherapy resistance and tumorigenesis [2]. Transition from anaplasic astrocytoma to glioblastoma malignant evolution [3] and intrinsic radioresistance [4] are promoted by protein kinase B (Akt) activation, which is also a negative prognosis factor [5]. Glycogen synthase kinase-3β (GSK-3β) and p53, protein substrates downstream of the PI3K/Akt pathway, also regulate cellular sensitivity/resistance to cancer chemotherapy and are unregulated in glioblastoma multiform [6,7].

Doxazosin (2-{4-[(2,3-Dihydro-1,4-benzodioxin-2-yl)carbonyl]piperazin-1-yl}-6,7-dimethoxyquinazolin-4-amine) is a quinazoline compound and a selective α1-adrenoceptor antagonist widely used for treatment of high blood pressure and urinary retention related with benign prostatic hyperplasia [8]. Early studies showed doxazosin induced apoptosis in murine prostatic stromal and epithelial cells [9,10] and on urothelial cancer [11], pituitary adenoma [12], breast cancer [2] and human glioblastoma cells (U87-MG) [13]. Sakamoto et al. suggested that early administration of doxazosin may be useful in preventing clinical prostate tumor formation and supressing metastasis of human prostate cancer [14].

Many studies have focused on cytotoxic effects of doxazosin on cell death in tumor cells, but not in neural non-tumor cells. Moreover, chemotherapeutics used in glioma treatment have poor permeability through the blood brain barrier and short half-lives. Due to its physicochemical characteristics, doxazosin is able to permeate the blood-brain barrier [15] (BBB) and its relatively long half-life provides basis for once-daily dosing, which is a therapeutic advantage [16].

Here we show that doxazosin has low neurotoxicity and induces cell death and G0/G1 phase arrest on C6 and U138-MG glioblastoma cells. When compared with the tyrosine kianse inhibitor Lapatinib, doxazosin appears to be a more potent antiglioma agent. We demonstrated that doxazosin’s antitumoral effects are due to downregulation of Akt and upregulation of GSK-3β and p53, in addition to activation of caspase 3. We also observed that doxazosin’s effects on the Phosphatidylinositol 3-Kinase/AKT pathway were similar as LY294002 (PI3K specific inhibitor).

## Materials and Methods

### Chemicals and materials

Cell culture medium and fetal bovine serum (FBS) were obtained from Gibco-Invitrogen (Grand Island, NY, USA). Doxazosin was obtained from Sigma Chemical Co (St. Louis, MO, USA). All other reagents were purchased from Sigma Chemical Co. (St. Louis, MO, USA) or Merck (Darmstadt, Germany). All chemicals and solvents used were of analytical or pharmaceutical grade.

### Cell culture

C6 rat (passage number 20–25) and U138-MG human glioma cell lines were obtained from American Type Culture Collection (Rockville, Mariland, Md., USA). C6 and U138-MG cells were grown and maintained in Dulbecco’s Modified Eagle’s Medium (DMEM, Gibco-Invitrogen, Grand Island, NY, USA) supplemented with 5% and 10% (v/v) FBS (Gibco-Invitrogen, Grand Island, NY, USA), respectively, and containing 2.5 mg/mL of Fungizone^®^ and 100 U/L of gentamicine (Shering do Brasil, São Paulo, SP, Brazil). Cells were kept at 37°C, in an atmosphere of 5% CO_2_.

### Ethics statement

All animal procedures were approved by the local animal ethics comission (Comissão de Ética no Uso de Animais/Universidade Federal do Rio Grande do Sul—CEUA/UFRGS, under project number 20.005) and follows national animal rights regulations (Law 11.794/2008), the National Institute of Health Guide for the Care and Use of Laboratory Animals (NIH publication No. 80–23, revised 1996) and Directive 2010/63/EU. We further attest that all efforts were made to minimize the number of animals used and their suffering.

### Primary astrocyte culture

Primary astrocyte culture from Wistar rats was prepared as previously described [17]. Newborn Wistar rats (1–2 days-old) were maintained in a ventilated room at constant temperature, in breeding cages with their mother, on a 12h light/dark cycle. The newborn rats were decapitated, their cerebral cortices were removed and mechanically dissociated in Ca²+ and Mg²+ free balanced salt solution, pH 7.4, containing (in mM): 137 NaCl; 5.36 KCl; 0.27 Na_2_HPO_4_; 1.1 KH_2_PO_4_ and 6.1 glucose. Cortices were cleaned of meninges and mechanically dissociated by sequential passage through a Pasteur pipette. After centrifugation at 1400 rpm for 5 min, the pellet was resuspended in DMEM (pH 7.6) supplemented with 8.39 mM HEPES, 23.8 mM NaHCO_3_, 0.1% amphotericin, 0.032% gentamicin and 10% Fetal Calf Serum (FCS). Cultures were maintained in DMEM containing 10% FCS in 5% CO_2_/95% air at 37°C, allowed to grow to confluence, and used at 15 days *in vitro*.

### Organotypic hippocampal slice culture

Organotypic hippocampal slice cultures were prepared according to the method of Stoppini [18] with modifications [19]. Wistar rats (6–8 days-old) were maintained in a ventilated room at constant temperature, in breeding cages with their mother, on a 12h light/dark cycle. The rats were decapitated, their hippocampi were removed and 400μM thick slices were prepared using a Mcllwain tissue chopper in ice-cold Hank’s balanced salt solution (HBSS), pH 7.2. Slices were placed on MiIlicell^®^ culture membranes and the inserts were transferred to a six-well culture plate. Each well contained 1mL of tissue culture medium consisting of Minimum Essential Media (MEM) with 25% of HBSS and 25% of horse serum supplemented with 36 mM glucose, 25 mM HEPES, 4 mM NaHCO_3_, 1% Fungizone^®^ and 0,1 mg/mL gentamicine, pH 7,3. The cultures were kept in an incubator 37°C and 5% of CO_2_ for 14 days.

### Culture treatments

C6 and U138-MG glioma cells were seeded in culture media and grown for 24 hours. Doxazosin was dissolved in 20% ethanol/milli-Q^™^ water (vehicle) and C6 cells were treated with concentrations ranging from 30 μM to 300 μM. U138-MG cells were treated with drug concentrations ranging from 5 μM to 75 μM. Primary astrocyte cultures and organotypic slice cultures were treated with doxazosin for 48 hours at 30–250 μM. Lapatinib was dissolved in DMSO and cells were treated with 500 nM.

### MTT assay

C6 and U138-MG glioma cells were plated in 96-well plates at 10³ cells/well and grown for 24 hours. Following treatment, cells were incubated with 0,5 mg/mL MTT (3-(4,5-methylthiazol-2-yl)-2,5-diphenyltetrazolium bromide) for 1 h. The solution was then removed from the precipitate and the formazan product in the cells was solubilized by adding DMSO. Absorbance was read by an ELISA plate reader (Biochrom Anthos Zenyth 200 Microplate Reader) at 490 nm.

### LDH assay

Cell death was evaluated by measuring the activity of lactate dehydrogenase (LDH, E.C.1.1.1.27). After treatment, U138-MG and C6 cell culture medium was collected and LDH activity was determined by an enzymatic colorimetric reaction (Cytotoxity Detection Kit—LDH, Roche Applied Science). Absorbance was measured at 490 nm.

### Sulforhodamine B assay

Sulforhodamine B (SRB) assay was used for cell density determination [20]. After treatment with doxazosin, cells were washed with Phosphate Buffered Saline (PBS) and fixed with PBS/FORMOL 4% for 15 minutes. Fixed cells were stained with SRB. Subsequently, wells were washed with deionized water to remove unbound stain. Culture plates were air dried and protein-bound SRB was solubilized in 1% SDS. Absorbance was measured by an ELISA plate reader (Biochrom Anthos Zenyth 200 Microplate Reader) at 515 nm.

### Flow Cytometry

Cell death was analyzed by flow cytometry. For C6, U138-MG and primary astrocyte cultures, both floating and trypsinized adherent cells were collected. Organotypic hippocampal slices were dissociated in PBS containing 1% collagenase, 1% DNase, and 0.2% trypsine and filtered through a 40 μm membrane (Millipore). Annexin-V FITC/propidium iodide (PI) double stain kit was used, following the manufacturer’s instructions (Invitrogen, Grand Island, NY, USA). Samples were incubated in binding buffer containing Annexin-V FITC and PI for 15 min in the dark at room temperature.

For cell cycle analysis, C6 and U138-MG cells were seeded in 6-well plates (3x10^4^ cells/well). After treatments, cells were washed with PBS, trypsinized and counted. Cells were centrifuged at 400 x g for 5 min and resuspended (10^6^ cells/mL) in PBS containing RNAse (100 μg/mL) and PI (5 μg/mL) for 15 min at room temperature.

For analysis of cleaved caspase 3, C6 and U138-MG cells were trypsinized and centrifuged at 400 x g for 5 min. Cells were resuspended in PBS containing 0,1% Triton X-100 and mouse anti-cleaved caspase 3 antibody (1:100; Cell Signaling), for 30 min at room temperature. Then, secondary antibody anti-mouse Alexa Fluor 488 (1:100, Invitrogen) was added and, after incubation for 30 min, fluorescence intensity was analyzed by flow cytometry.

Data acquisition was done by flow cytometry using a FACS Calibur cytometry system and Cell Quest software (BD Bioscience, Mountain View, CA, USA). Data obtained was analyzed with FCS Express 4 Software (De Novo Software, Los Angeles, CA, USA).

### Western blotting

After treatments, cells were homogenized in lysis buffer (4% sodium dodecyl sulfate (SDS), 2 mM EDTA, 50 mM Tris). Protein concentration was determined by the Lowry method. Proteins were resolved (75 μg per lane) on 10 or 12% SDS-PAGE and transferred to nitrocellulose membranes (Hybond™ ECL™ nitrocellulose membrane, Amersham Biosciences™, Fryeburg, Germany) using a semi-dry transfer apparatus (Bio- Rad™, Trans-Blot SD, Hercules™, CA, USA). Membranes were incubated for 60 minutes at 4°C in blocking solution (Tris-buffered saline containing 5% powdered milk and 0.1% Tween-20, pH 7.4). and incubated overnight with specific antibodies. Primary antibodies (Cell Signaling Technology™, Beverly, MA, USA) against the following proteins were used: anti-p-AKT_Ser473_ and anti-AKT (1:1000), anti-p-GSK-3β_Ser9_ and anti-GSK-3β (1:1000), anti-p-p53_Ser15_ (1:1000), anti-β-actin (1:1000). Membranes were then incubated with horseradish peroxidase conjugated anti-rabbit antibody (1:1000; Amersham Pharmacia Biotech, Piscataway, NJ, USA). Chemiluminescence (ECL, Amersham Pharmacia Biotech™) was detected using X-ray films (Kodak X-Omat™, Rochester, NY, USA).

### Statistical analysis

Data are expressed as means±SEM. All results are representative of at least 4 independent experiments. Analysis of variance (ANOVA) was applied to the means to determine statistical differences between experimental groups. Post hoc comparisons were performed by Tukey test. Differences between mean values were considered significant when p<0.05.

## Results

### Evaluation of doxazosin toxicity on non tumoral tissue/cells

One of the problems of current cancer therapy is lack of specificity and selectivity of certain drugs used for tumor treatment [21]. In order to evaluate the drug toxicity on non-tumoral neural tissue, we exposed primary astrocyte cultures and organotypic hippocampal slice cultures to doxazosin for 48 hours.

**Fig 1:**
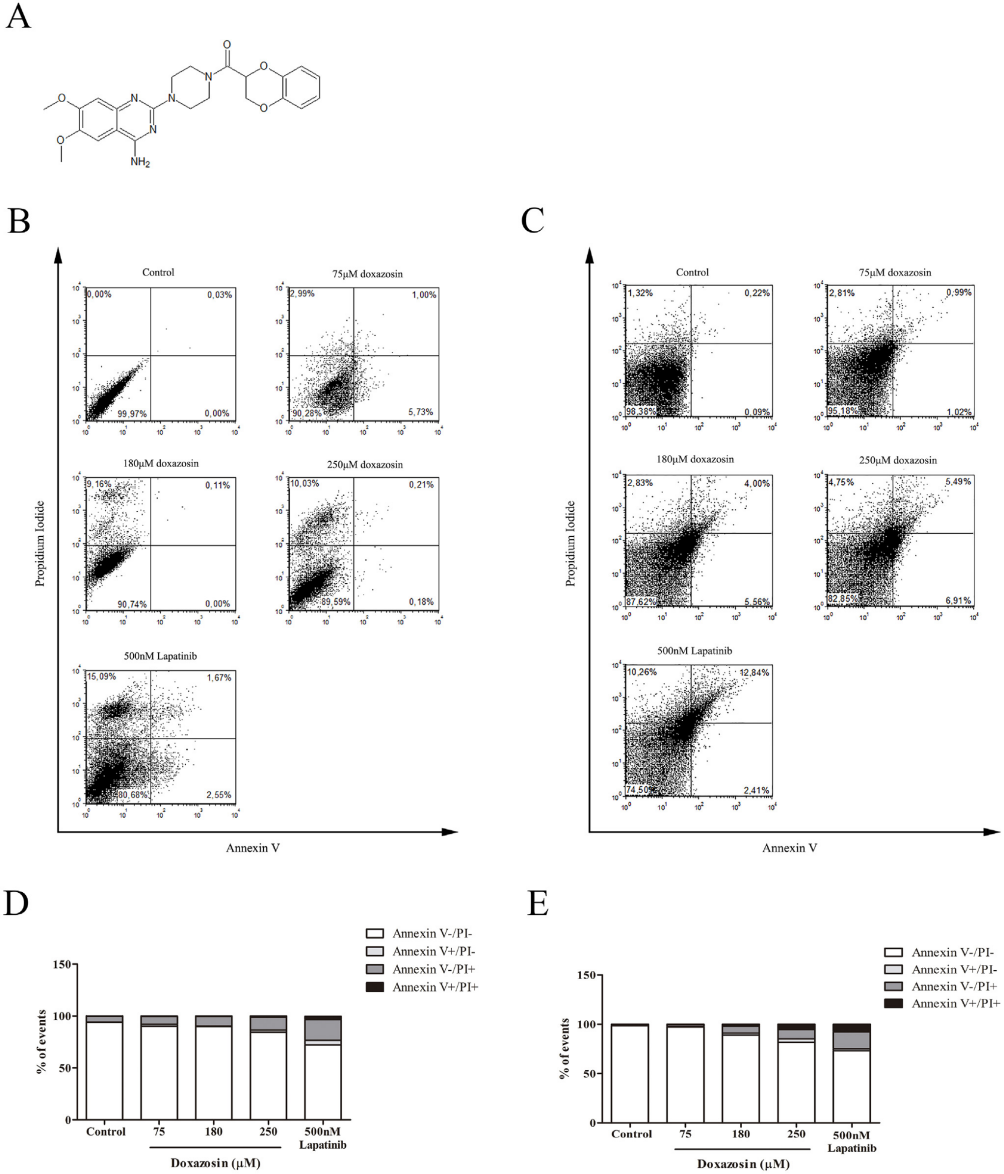
Effects of doxazosin and Lapatinib on non-tumor cells stained with Annexin V (AnV) and Propidium Iodide (PI). **(A)** Doxazosin’s molecular structure. **(B)** Dot plot and graph **(D)** of primary astrocytes cultures after treatment with doxazosin and Lapatinib for 48h. **(C)** Dot plot and graph **(E)** of organotypic hippocampal cultures treated with doxazosin and Lapatinib for 48h. Data are represented as means (n = 4).

Initially, we tested a concentration curve (30–250 μM) of doxazosin, with intermediate concentrations obtained from the literature [22]. In primary astrocyte culture (Fig 1 and S1 Table), 250 μM doxazosin increased total cell death (15.62%±2.674), while at concentrations of 75 and 180 μM was not observed significant cell death. Lapatinib (500 nM) caused total cell death of 27.61%±1.218, inducing necrosis in 20.07%±1.261. The toxicity on non-tumoral astrocytic cells by Lapatinib was higher than doxazosin 250 μM.

In organotypic cultures (Fig 1 and S1 Table), doxazosin caused cell death of 10.82%±1.232 at 180 μM and 18.27%±1.346 at 250 μM. Lapatinib (500 nM) caused cell death in 26.86%±1.885 of cells, inducing necrosis in 17.28%±6.134 of cells. In non-tumoral neural tissue, Lapatinib cell death induction also was higher than doxazosin.

In relation to the distribution and type of cell death observed on organotypic cultures, doxazosin induced apoptosis and necrosis at 250 μM mostly on CA1 region (S1 Fig). Lapatinib induced apoptosis and necrosis on CA1 and mostly on the dentate gyrus (DG) regions.

### Antiglioma activity of doxazosin

**Fig 2:**
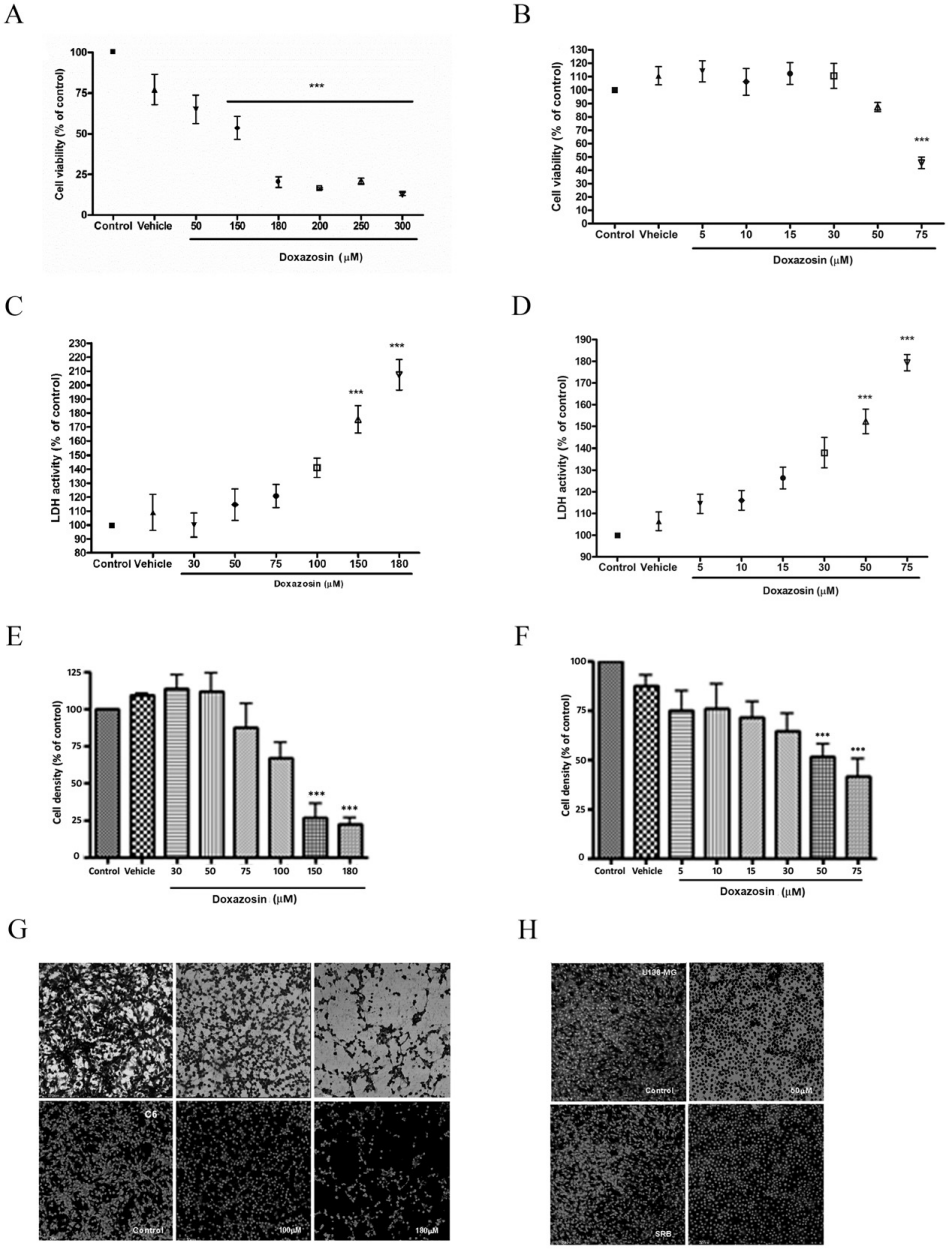
Citotoxicity of doxazosin on C6 (A,C,E,G) and U138-MG (B,D,F,H) cells after treatment for 48 h. MTT assay of C6 **(A)** and U138-MG **(B)**. LDH activity of C6 **(C)** and U138-MG **(D)**. SRB assay of C6 **(E)** and U138-MG **(F)**. Data are represented by means±SEM (n = 4). ***p<0.001 compared to respective control, ANOVA followed by Tukey’s test. Confocal microscopy of C6 **(G)** and U138-MG **(H)**. Photomicrographs are on top and SRB staining in the bottom. Magnification: 10X + 2,5.

C6 and U138-MG cells were treated with a concentration curve of doxazosin for 48 hours, based on concentrations used for non-tumor cells. In C6 cells, we observed a significant reduction of cell viability with treatments from 150 μM to 300 μM of doxazosin for 48 h (Fig 2). Doxazosin increased LDH activity in the incubation medium of C6 cells when treated with 150 and 180 μM (Fig 2). In agreement with these results, cell density of C6 cells decreased to approximately 25% when treated with doxazosin 150 and 180 μM for 48 h (Fig 2).

For U138-MG cells, after 48 hours of treatment we observed a significant reduction on the percentage of viable cells in cultures treated with 75 μM of doxazosin (Fig 2). LDH activity increased in the incubation medium of U138-MG culture cells when treated with 50 and 75 μM doxazosin (Fig 2). Also at these concentrations, doxazosin decreased cell density to approximately 50% (Fig 2), supporting the results above. All together, these cytotoxicity assays suggest an antiglioma effect of doxazosin in C6 and U138-MG cell lines.

### Doxazosin induced cell death by necrosis and caspase-dependent apoptosis in human and rat glioblastoma cell lines

**Fig 3:**
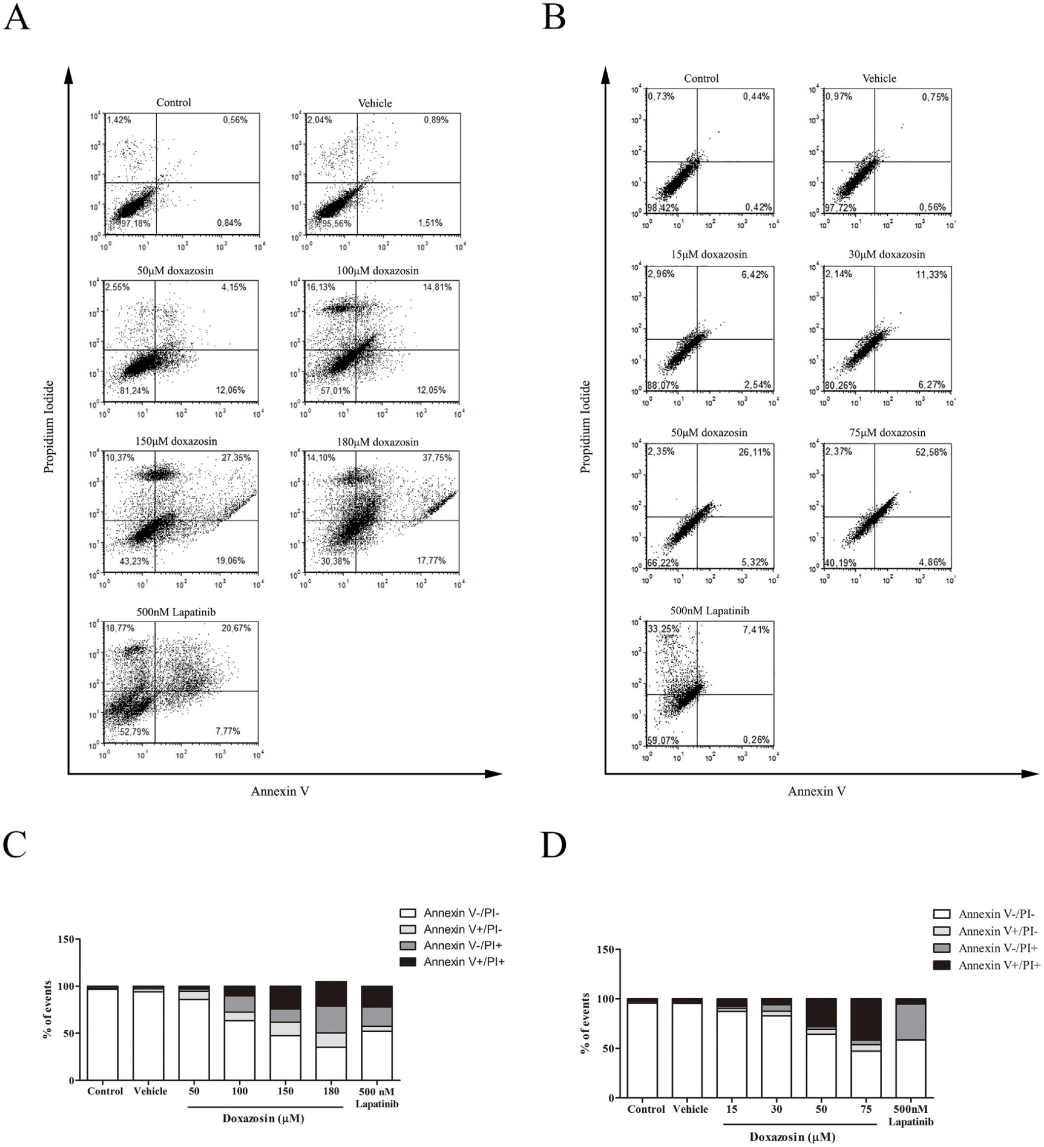
Effects of doxazosin and Lapatinib on glioma cells stained with Annexin V (AnV) and Propidium Iodide (PI). **(A)** Dot plot and graph **(C)** of C6 cells after treatment with doxazosin and Lapatinib for 48h. **(B)** Dot plot and graph **(D)** of U138-MG cells treated with doxazosin and Lapatinib for 48 h. Data are represented as means (n = 4).

Doxazosin induced necrosis and apoptosis in C6 cells after 48 h of treatment (Fig 3, S2 Table and S2 Fig). At concentration of 100 μM, the drug caused 36.68%±5.045 of cell death and this toxic effect increased with higher drug concentrations, reaching 69.71%±2.503 at 180 μM. At 180 μM, doxazosin caused early apoptosis in approximately 14.89%±1.581 of cells and late apoptosis in approximately 26.20%±4.860. Necrosis occurred in 28.62%±6.373. While Lapatinib caused cell death in 47.96%±0.975 of C6 cells after 48 h, triggering necrosis in 20.56%±1.591 and late apoptosis in 22.01%±1.548 of cells. Lapatinib caused cell death on C6 cells to a less extent than doxazosin.

In U138-MG cells, doxazosin induced cell death in 35.9%±3.356 of cells at 50 μM and 52.69%±5.709 at 75 μM after 48 h (Fig 3 and S2 Table). Doxazosin 75 μM triggered mostly late apoptosis (41.86%±5.589) in U138-MG cells. Lapatinib caused cell death in 41.44%±0.466 of cells, and triggered mostly necrosis (36.13%±1.733) (Fig 3 and S2 Table).

**Fig 4:**
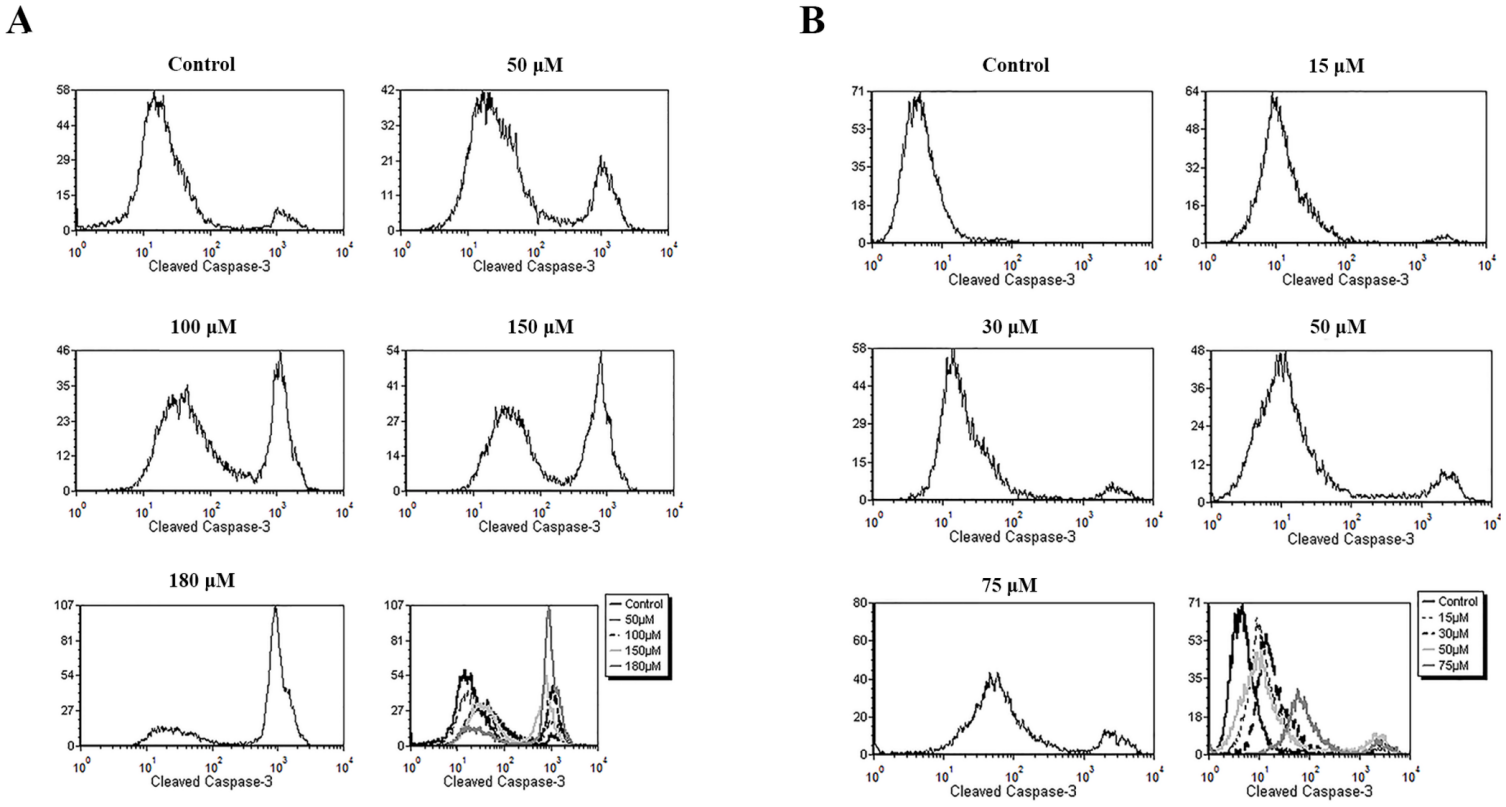
Flow cytometry of cleaved caspase-3 of C6 (A) and U138-MG (B) glioma cells.

After 48 h of treatment, doxazosin increased activation of caspase 3 in C6 and U138-MG (Fig 4) cell lines and this was proportional to increase in drug concentration. These results of cleaved caspase-3 levels confirm induction of apoptosis by doxazosin.

### Doxazosin caused G0/G1 phase arrest on C6 and U138-MG glioblastomas

**Fig 5:**
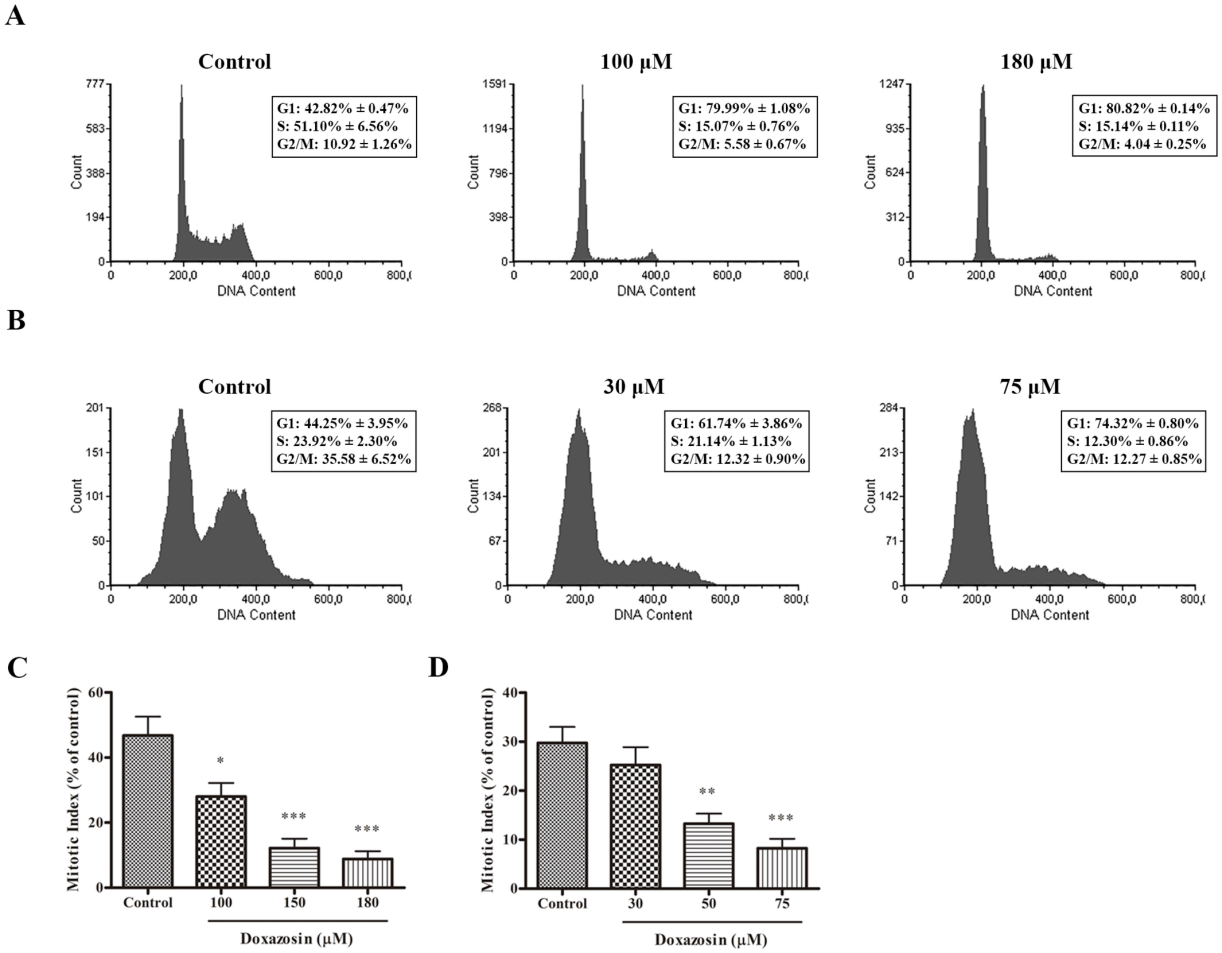
Cell cycle analysis (A,B) and mitotic index (C,D) of glioma cells treated with doxazosin for 48 h. (**A**) Cell cycle of C6 cells and (**B**) U138-MG cells. (**C**) Mitotic index of C6 cells and (**D**) U138-MG cells. Data are represented by means±SEM (n = 4). *p<0.05, **p<0.01, ***p<0.001 compared to respective control, ANOVA followed by Tukey’s test.

We next sought to analyze doxazosin’s effect on cell cycle progression and mitotic index. As shown in Fig 5, after 48 h of treatment of C6 cells, doxazosin caused cell cycle arrest at the G0/G1 phase at concentrations of 100 and 180 μM compared to control. We analyzed the mitotic index of C6 cells in order to assess proliferation (Fig 5). Doxazosin decreased the mitotic index in the concentrations of 100, 150 and 180 μM after 48h of treatment, in agreement with cell cycle results.

In U138-MG cells, doxazosin caused cell cycle arrest in G0/G1 phase at concentrations of 30 and 75 μM and decreased mitotic index in the concentrations of 50 and 75 μM after 48 h of treatment in U138-MG cells (Fig 5).

### PI3K/Akt pathway: a possible doxazosin target

**Fig 6:**
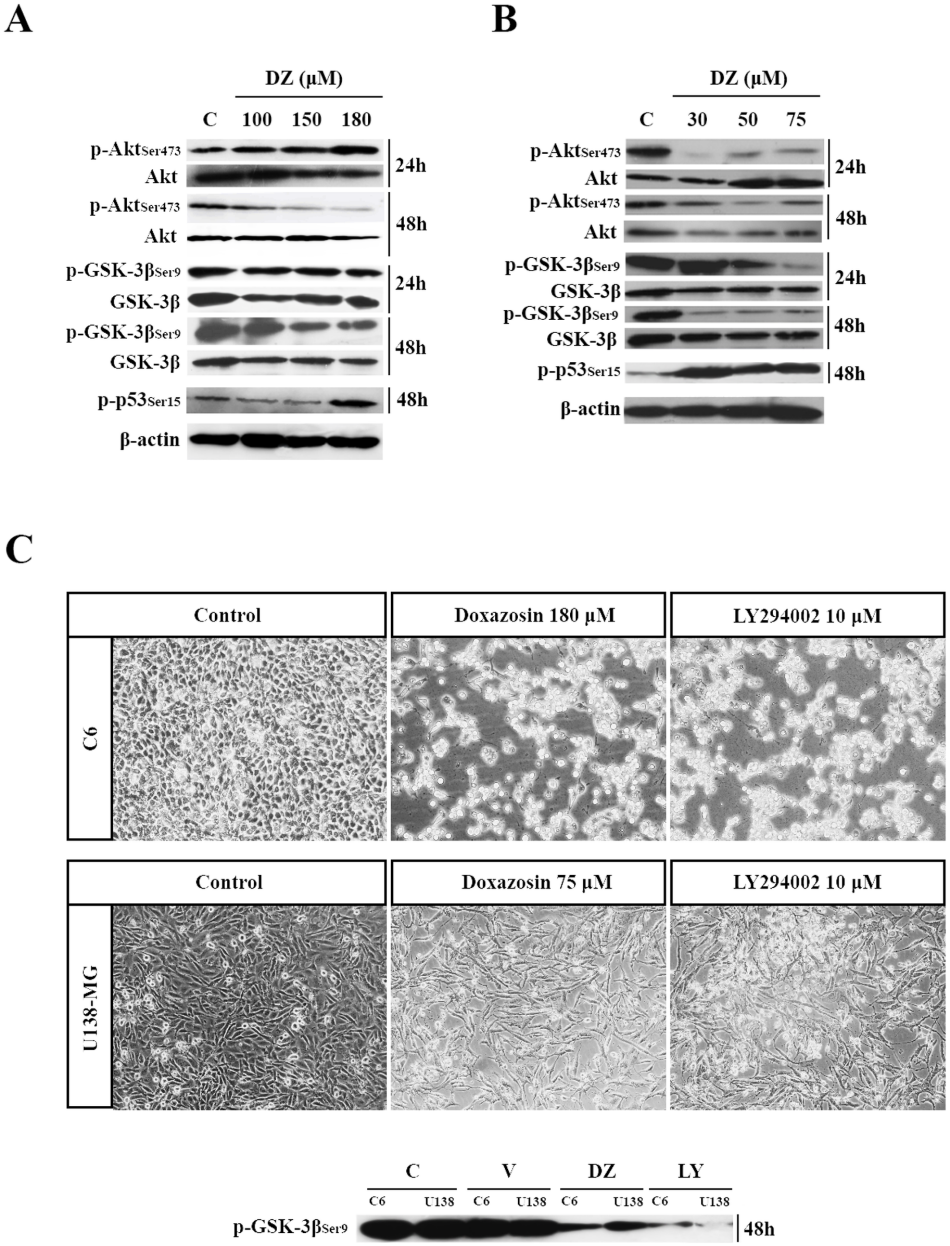
Western blotting of C6 (A) and U138-MG (B) cells after treatment with doxazosin for 48h (n = 4). β-actin was used as a loading control. **(C)** Effect of doxazosin on cell death and GSK-3β Ser9 phosphorylation compared with LY294002 (**LY**) on glioma cells. Magnification: 40X. **C**–control; **V**–vehicle; **DZ**–doxazosin. Data are represented as means (n = 4).

Next, we analyzed activation of proteins involved with apoptosis, cell cycle and glioma malignancy in rat and human glioblastoma cells. The phosphorylation levels of p-AKT_Ser473_, p-GSK-3β_Ser9_ and p-p53_Ser15_ were analyzed for their role in cell survival, proliferation and apoptosis regulation. Doxazosin was added for 24 and 48 h. As shown in Fig 6, p-Akt_Ser473_ levels decreased at 150 and 180 μM doxazosin in C6 cells after 48 h.

Additionally, we evaluated whether Akt downstream target GSK-3β was affected. As shown in Fig 6, the treatment of C6 cells with doxazosin, 150 and 180 μM, decreased p-GSK-3β_Ser9_ levels after 48 h. We also observed an increase of phosphorylation levels of p-p53_Ser15_ after 48 h of treatment with doxazosin 180 μM in C6 cells (Fig 6).

In U138-MG cells, treatment with doxazosin during 24 h and 48 h at concentrations of 30, 50 and 75 μM, decreased p-Akt_Ser473_ levels (Fig 6). By other hand, after 24 h only the treatments at concentrations of 50 and 75 μM of doxazosin were able to decrease the p-GSK-3β_Ser9_ levels, while after 48 h this decrease was maintained and we also observed a decrease at 30 μM of doxazosin treatment (Fig 6). Regarding to the effect of doxazosin on the p53 protein in U138-MG cells, it was observed an increase in p-p53_Ser15_ at the concentrations of 30, 50 and 75 μM (Fig 6). We analyzed immunocontent of these same phosphorylated proteins at earlier times and did not observe any differences (data not shown).

In order to analyze whether the PI3K signaling pathway by Akt activation and GSK-3β inactivation was involved in the effect of doxazosin, we carried out this experiment using LY294002, a specific inhibitor of PI3K [23]. Activated PI3K phosphorylates its downstream target Akt. Phosphorylated Akt directly affects GSK-3β by phosphorylating it at Ser9 and thus inhibiting its activity. LY294002 induced C6 and U138-MG cell death in 48 h and inhibited GSK-3β_Ser9_ phosphorylation in the same manner as doxazosin (Fig 6). This result indicates a possible effect of doxazosin on the PI3K/Akt pathway on glioma cells.

## Discussion

Current anticancer drugs have limitations by presenting cytotoxicity, low specificity and the prolonged use may result in lethal damage to healthy cells. Therefore, chemotherapeutic treatment of tumors in the central nervous system is associated with severe systemic side effects and therefore affecting patients quality of life [24]. Glioblastoma is a radio- and chemo-resistant cancer. This makes it necessary to increase chemotherapy dosage or add more drugs for treatment, which increases toxicity on non-tumor cells. Tumor surgical resection, in the case of glioblastomas, might compromise vital brain areas due to the cancer’s aggressive and infiltrative characteristics [24].

Temozolomide (TMZ) is considered the standard therapy for treatment of glioblastoma multiforme. However TMZ must be administered at high doses systemically in order to achieve therapeutic levels in the brain, mainly due to its low half-life (about 1.8 h in plasma) [25]. It has been approved by the FDA in the USA for the treatment of GBM, showing a median survival of just 5.8 months [26]. Furthermore, prolonged systemic administration is associated with side effects such as nausea, vomiting, fatigue and headache.

Additionally, the central nervous system has another limitation regarding treatment: nerve tissue has a low rate of cell division and loss of non-tumor cells greatly affects the well-being of patients. For these reasons, the search for new therapeutic strategies and chemotherapeutics is necessary to improve effectiveness of glioma treatment, while preserving the quality of life of patients. Doxazosin is a FDA approved drug in the USA for treatment of hypertension and symptoms of benign prostatic hyperplasia, with mild side effects, like dizziness and hypotension [22,27]. However, doxazosin toxicity on nervous non-tumor tissue has never been evaluated so far.

In astrocytes, doxazosin increased total cell death only at the high concentration of 250 μM when compared to control. In organotypic cultures, doxazosin caused cell death at 180 and 250 μM concentrations when compared to control. Although there was no statistical difference between the types of cell death, doxazosin showed a tendency towards induction of necrosis and late apoptosis. These results show that doxazosin triggers different cell responses depending on its concentration and the best range of concentrations for further tests are below 250 μM.

We also tested Lapatinib citotoxicity on primary astrocytes and organotypic cultures, and compared its antitumoral effect with doxazosin on C6 and U138-MG glioma cells. Lapatinib is a tyrosine kinase inhibitor, which has a quinazolinic ring in its chemical structure. Compounds based on quinazoline scaffolds can target a range of kinases with varying degrees of selectivity [28]. Doxazosin also presents a quinazoline scaffold on its chemical structure. Lapatinib was used in this study as a comparison with doxazosin because they present similar chemical structure [29]. Reports show Lapatinib effective inhibition of human breast and prostate cancer cell lines, and in U87-MG and M059K glioblastoma cell lines [30]. Here we showed that Lapatinib 500 nM is more cytotoxic than doxazosin 250 μM on primary astrocytes and hippocampal organotypic cultures.

We observed that in hippocampal organotypic cultures doxazosin induced apoptosis on DG and CA1 cells, but necrosis was limited to CA1 area, possibly due to delayed cell death and DG’s capability of apoptosis induction for tissue remodeling purposes [31]. Lapatinib also induced apoptosis and necrosis on both regions, but necrosis was more pronounced in the DG region. CA1 neurons react differently after lesions than DG cells, mainly because they present more dendritic remodeling and present high abundance of NMDA (*N*-methyl-D-aspartate) receptors, which could lead to glutamate excitotoxicity [32].

Several studies have demonstrated that doxazosin’s concentrations responsible for cell death induction vary depending on the origin of the cells, the type of cancer and even between different cell lines of the same type of tumor [22,33]. On prostate cancer cells, for example, maximum concentration of doxazosin used is 100 μM [22,34]. On the other hand, colon cancer SW-480 cells and bladder cancer HTB1 cells were less sensitive to this concentration of doxazosin [22]. HeLa cells showed high sensitivity to doxazosin, while HepG-2 and MCF-7 cells showed an IC50 of around 200 μM [35].

We observed that C6 and U138-MG cell lines had different vulnerability against doxazosin. Results showed that doxazosin induced apoptosis and necrosis on C6 cells and only apoptosis on U138-MG cells. When compared with Lapatinib, doxazosin showed to be more effective in inducing apoptotic cell death in both C6 and in U138-MG cells. Although it has been observed that treatment with TMZ induces a decrease in cell viability in various glioma lines, it is well described that U138-MG lineage is resistant to this treatment [36]. Our results showed that doxazosin was able to induce apoptosis in both cell types, C6 and U138-MG. Differences observed between cell lines on cytotoxicity assays and types of cell death could be due to: different species, varying doubling times or characteristics and mutations between cell lineages. C6 cells have a doubling time of approximately 25–30 hours [37], while U138-MG cells’ is 70 hours [38]. C6 cells proliferate faster, being more aggressive than U138-MG. Moreover, U138-MG cells have higher expression of the pro-apoptotic protein Bax, being more sensitive to apoptosis [33]. This could explain the different concentrations to which doxazosin was more effective (50 and 75 μM for U138-MG cells and 150 and 180 μM for C6 cells).

Doxazosin’s effective concentrations used on this study on rat C6 cells (150 and 180 μM) exceeds the limits used for most cancer cells on the literature [22,34]. For the human cell line U138-MG, effective doxazosin concentrations are 50 and 75 μM, which is in accordance with what was used in the literature for other cancer cells. Future studies, however, are necessary to determine if this *in vitro* therapeutic concentrations of doxazosin are effective against glioblastoma *in vivo* models.

In addition to inducing cell death, doxazosin decreased proliferation, shown by cell cycle arrest in G0/G1 phase and decreased mitotic index. On human LNT-229 and U87-MG GB cells, doxazosin also induced G0/G1 cell cycle arrest and concentration-dependent apoptosis [13]. Several *in vitro* and *in vivo* studies show that the main mechanism underlying doxazosin-induced apoptosis is inhibition of receptor-mediated signaling [39,40,41]. Therefore, we suggest that those effects and the apoptosis induction may be related with the decrease of Akt phosphorylation/activation, which possibly affected the Akt downstream targets, GSK-3β and p53. In PC-3 cells, doxazosin induced apoptosis in part through downregulation of Akt cell signaling. Akt plays a pivotal role regulating cell growth and survival in cancer cells. This fact justifies the pharmacological use of doxazosin and derivatives, which possess quinazolinic rings, for the development of a new class of apoptosis-inducing agents, which blocks activation of Akt protein [42].

Moreover, doxazosin was shown to suppress PI3K and Akt phosphorylation on ovarian carcinoma cells [41]. The authors of this study suggest the hypothesis that doxazosin inhibits PI3K/Akt activity. GSK-3β is involved in the processes of apoptosis and cell cycle, as well as p53. One of the most important regulatory mechanism acting on GSK-3β is phosphorylation of Ser9, which inhibits the protein. Several kinases are able to phosphorylate this protein, including Akt [43]. Here we found that doxazosin, possibly through Akt inhibition, decreased GSK-3β Ser9 phosphorylation. Also we observed that doxazosin cell cycle arrest and the decrease on proliferation of C6 and U138-MG glioma cells appears to be mediated by GSK-3β dephosphorylation/activation.

In cancer, GSK-3β has been reported as a “tumor suppressor” by repressing neoplastic transformation and tumor development[44]. GSK-3β also appears to be involved in chemotherapy resistance. When treated with lithium, which is a GSK-3β inhibitor, hepatoma cells became resistant to etoposide and camptothecin. On the other hand, GSK-3β activation with LY294002 and with exogenous expression of Ser9 GSK-3β sensitizes hepatoma cells to apoptosis induced by those drugs [45]. LY294002 can also inhibit expression of p-Akt in cancer cells via PI3K suppression [46], which supports the induction of cell death on C6 and U138-MG cells shown here. Other reports show that GSK-3β activation enhances and sensitizes human breast cancer cells to paclitaxel, 5-fluorouracil, cisplatin, taxol and prodigiosin [43]. Also, other studies with GSK-3β inhibitors on GBM patients and two established cell lines (U251 and U87-MG) showed that inhibition of GSK-3β phosphorylation significantly reduced tumor invasiveness [6].

Another protein that we found altered by doxazosin treatment is p53. This is a tumor-suppressor protein that is activated upon various types of cellular stresses. The p53 protein is involved with apoptosis induction, with inhibition of cell cycle progression [47], as demonstrated here on C6 and U138-MG cells. In almost all types of cancer, p53 is frequently inactivated, being a central tumor-suppressor. The p53 protein can regulate and be regulated by Akt and also can interact with GSK-3β. It is well established that activation of Akt inhibits p53-mediated apoptosis [47].

The p53 gene is mutated in 30–50% of glioblastomas and this mutation confers increased malignancy and tumorigenicity to the cancer. Also it has been reported that p53 mutation decreases chemosensitivity of malignant gliomas to TMZ [7]. The U138-MG cell line has a TP53 mutation and is TMZ resistant [48]. Here we showed that doxazosin, unlike TMZ, decreased cell viability and induced apoptosis in U138-MG cells, and also increased p53 phosphorylation in these cells.

We also found that doxazosin activated caspase-3 on glioma cells. Caspase-3 can be activated through inhibition of Akt phosphorylation, and consequently leading to the inhibition of this kinase. When Akt is inactive, cells can undergo apoptosis through loss of mitochondrial membrane potential, leading to activation of caspase-9 and caspase-3, resulting in apoptosis [49].

**Fig 7:**
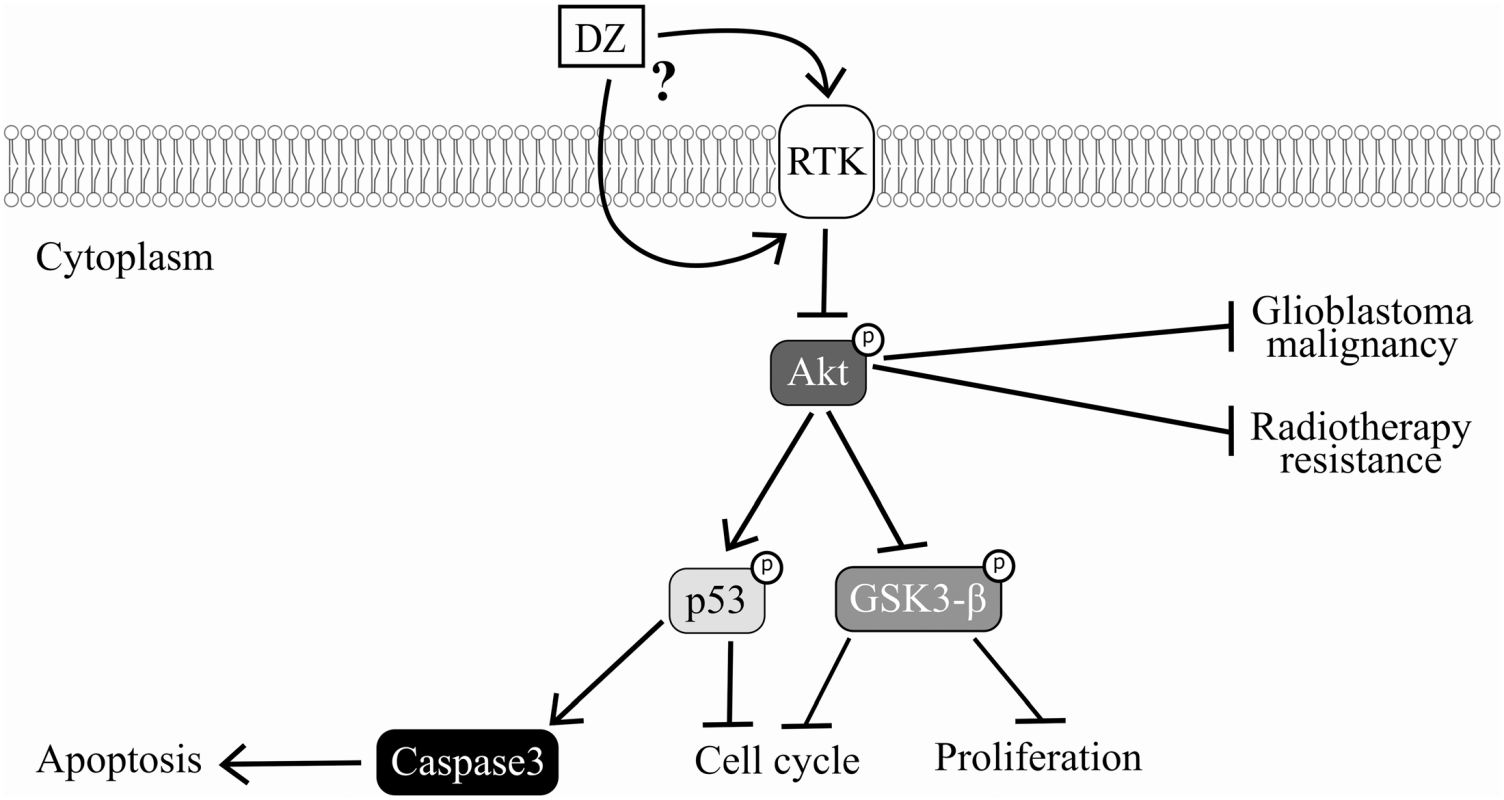
Suggested model of the effects of doxazosin on C6 and U138-MG glioma cells. Doxazosin promotes inhibition of PI3K/Akt pathway, which activates GSK-3β and p53. GSK-3β inhibits cell cycle progression and cell proliferation and p53 induce apoptosis via caspase-3 activation, and also inhibits cell cycle progression.

In summary, our results suggest that doxazosin induces caspase-dependent apoptosis, decreased mitotic index and induced cell cycle arrest on C6 and U138-MG glioma cells, and this effect could be mediated by the inhibition of Akt and the activation of GSK-3β and p53 proteins (Fig 7). Besides, we also show that doxazosin has low cytotoxicity on primary astrocytes and hippocampal organotypic cultures.

## Conclusions

Development of new effective therapeutic strategies for the treatment of brain tumors is essential to reduce mortality and morbidity of the disease. Characterization of new drugs that may act as an adjuvant to other anti-tumor therapies could be a very useful strategy for treating gliomas. Doxazosin’s pharmacology and safety profile is well-characterized in humans and its adverse-effect profile is acceptable [22], since the most frequent side-effects are dizziness and hypotension [50]. Moreover, Kyprianou and Benning [22] demonstrate that the therapeutic doses of doxazosin with antitumoral effect in mice (3 to 100 mg/Kg) are compared to intracellular doxazosin’s concentrations effective on human prostate cancer cells *in vitro* (100 μM). They showed *in vivo* efficacy studies in which doxazosin treatment suppressed significantly the tumorigenic growth of prostate cancer xenographs in SCID mice. Chon et al. [9] and Vashist et al. [51] also demonstrated the in vivo functional significance of doxazosin’s action in a mouse model of prostate hyperplasia and in the persistent reduction of intimal hyperplasia in a rabbit model, respectively.

Therefore, we and others [13,22,41,52] suggest that doxazosin can become a new pharmacotherapy alternative for the treatment of cancer, especially gliomas, although more experiments are needed to further elucidate the mechanism of action of this drug on tumor cells and animal models.

## Supporting Information

S1 Fig Distribution of cell death on hippocampal slices after treatment for 48h. **(A)** Photomicrographs of organotypic hippocampal slice cultures stained with Annexin V and Propidium Iodide after treatment with doxazosin or Lapatinib for 48 hours. Magification: 40X. **(B)** Schematic representation of a hippocampal slice.
(TIF)

S2 Fig Cell death on C6 culture after treatment for 48h. Photomicrographs of C6 glioma cells stained with Annexin V and Propidium Iodide after treatment with doxazosin or Lapatinib for 48 hours. Magification: 200X.
(TIF)

S1 File Methodology used for fluorescence microscopy analysis of cell death on organptypic hippocampal cultures, C6 and U138-MG cells. Describes the materials and methods used for fluorescence microscopy analysis of cell cultures.
(DOC)

S1 Table Descriptive statistics of percentage of cell death on neural non-tumor cultures. (DOC)

S2 Table Descriptive statistics of percentage of cell death on glioma cell lines. (DOC)
