# Supplementary figures and images for: Phosphatidylinositol 3-Kinase/AKT Pathway Inhibition by Doxazosin Promotes Glioblastoma Cells Death, Upregulation of p53 and Triggers Low Neurotoxicity
Source: PLoS One. 2016 Apr 28;11(4):e0154612. doi: 10.1371/journal.pone.0154612 (PMC4849739; doi:10.1371/journal.pone.0154612)

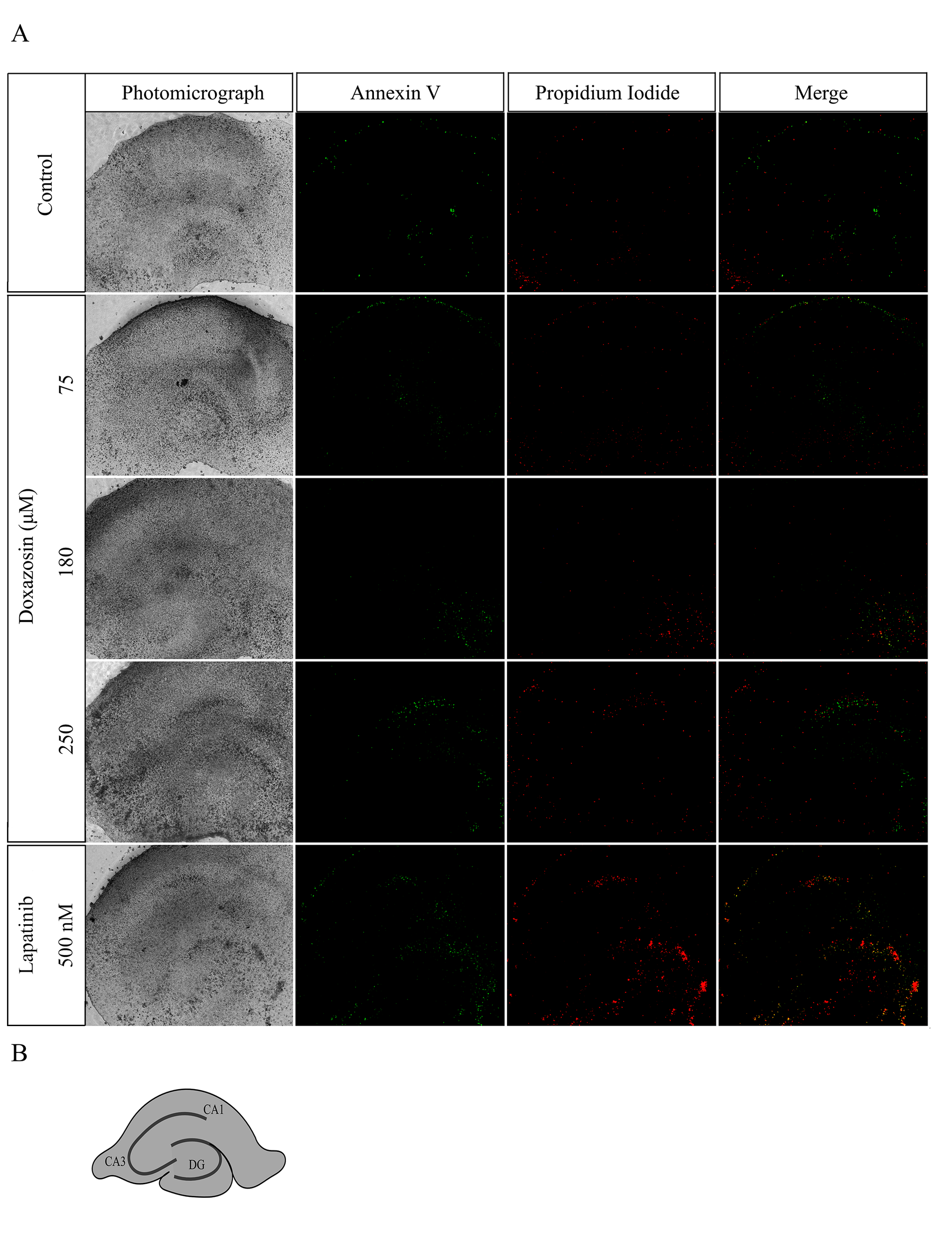

Supplement: S1 Fig — (A) Photomicrographs of organotypic hippocampal slice cultures stained with Annexin V and Propidium Iodide after treatment with doxazosin or Lapatinib for 48 hours. Magification: 40X. (B) Schematic representation of a hippocampal slice. (TIF) [file pone.0154612.s001.tif]

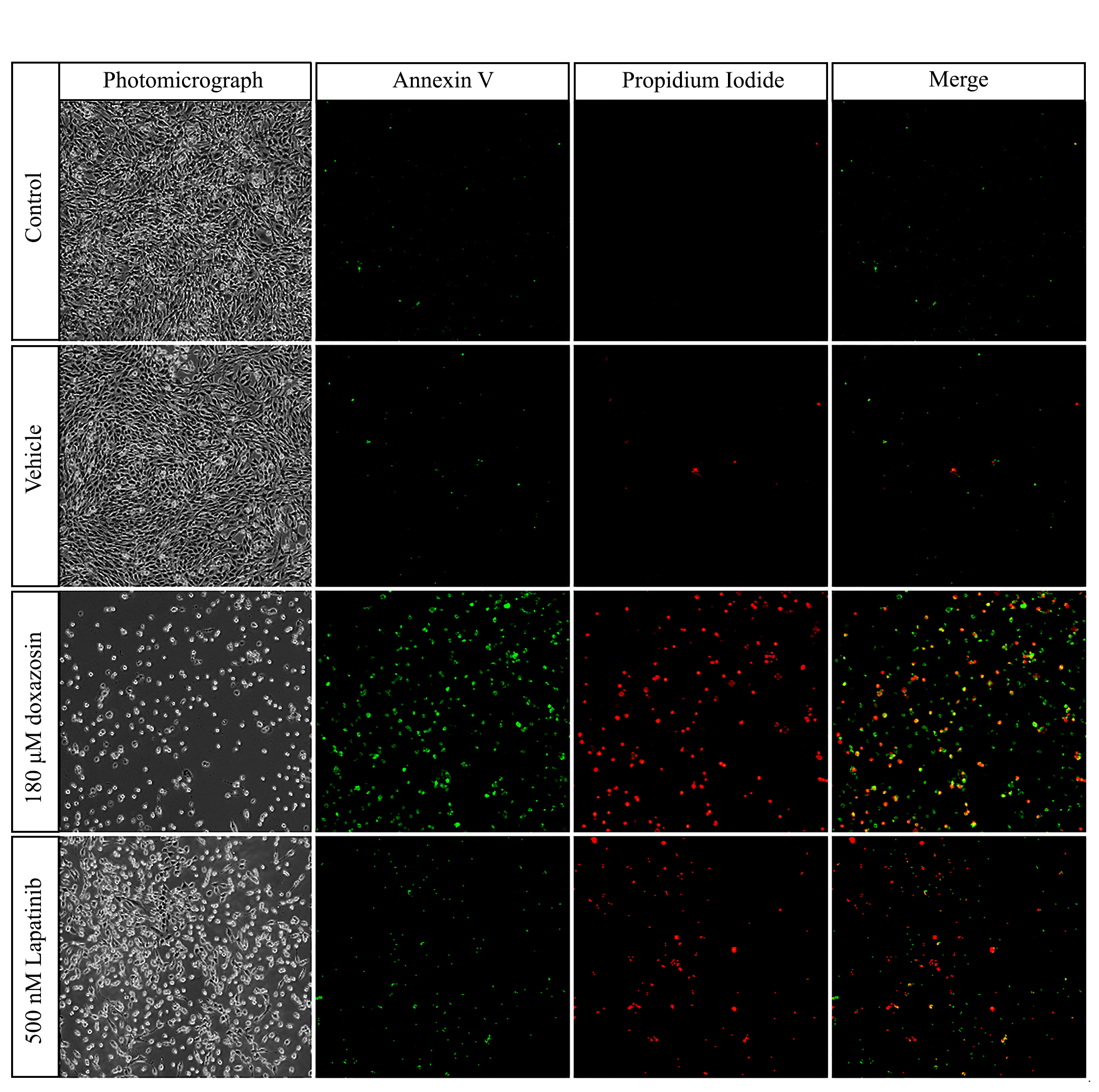

Supplement: S2 Fig — Photomicrographs of C6 glioma cells stained with Annexin V and Propidium Iodide after treatment with doxazosin or Lapatinib for 48 hours. Magification: 200X. (TIF) [file pone.0154612.s002.tif]
