# Supplementary material for: Phosphatidylinositol 3-Kinase/AKT Pathway Inhibition by Doxazosin Promotes Glioblastoma Cells Death, Upregulation of p53 and Triggers Low Neurotoxicity
Source: PLoS One. 2016 Apr 28;11(4):e0154612. doi: 10.1371/journal.pone.0154612 (PMC4849739; doi:10.1371/journal.pone.0154612)
Supplement: S1 File — Describes the materials and methods used for fluorescence microscopy analysis of cell cultures. (DOC) [file pone.0154612.s003.doc]

**Supplementary Materials and Methods**

***Fluorescence Microscopy***

To identify cellular death, Annexin-V fluorescein isothiocyanate (FITC)/propidium iodide (PI) double stain kit was used (Invitrogen, Grand Island, NY, USA). For C6, U138-MG and organotypic cultures, 2 µL of Annexin-V FITC and 1 µL of PI were added to 6 well plates containing 2 mL per well, and incubated for 15 min in the dark at 37°C. Annexin-V FITC and PI fluorescence were analyzed in an inverted microscope (Nikon Eclipse TE300). Images were captured using a digital camera connected to the microscope and analyzed using MacBiophotonics ImageJ software.
