# Supplementary material for: Phosphatidylinositol 3-Kinase/AKT Pathway Inhibition by Doxazosin Promotes Glioblastoma Cells Death, Upregulation of p53 and Triggers Low Neurotoxicity
Source: PLoS One. 2016 Apr 28;11(4):e0154612. doi: 10.1371/journal.pone.0154612 (PMC4849739; doi:10.1371/journal.pone.0154612)
Supplement: S1 Table — (DOC) [file pone.0154612.s004.doc]

**Supplementary Table 1** Descriptive statistics of percentage of cell death on neural non-tumor cultures.

|  | **AnV-/PI-** | **AnV+/PI-** | **AnV-/PI+** | | **AnV+/PI+** | **Total Cell Death** |
| --- | --- | --- | --- | --- | --- | --- |
| **Primary Astrocyte Culture** | | | |  | | |
|  |  |  |  | |  |  |
| **Control** | 93.98±1.386 | 0.22±0.0808 | 5.68±1.321 | | 0.14±0.054 | 6.03±1.393 |
| **Doxazosin** |  |  |  | |  |  |
| **75 µM** | 90.17±0.793 | 1.95±1.89 | 7.48±2.396 | | 0.41±0.298 | 9.83±0.793 |
| **180 µM** | 89.98±1.279 | 0.36±0.316 | 9.27±1.704 | | 0.40±0.265 | 10.03±1.278 |
| **250 µM** | 84.39±2.679^*^ | 2.08±0.990 | 12.44±1.466 | | 1.10±0.450 | 15.62±2.674 |
| **Lapatinib** |  |  |  | |  |  |
| **500 nM** | 72.13±0.998^***,##^ | 8.45±4.654 | 20.07±1.261^***^ | | 3.14±1.603 | 27.61±1.218 |
|  |  |  |  | |  |  |
| **Hippocampal Organotypic Culture** | | | |  | | |
|  |  |  |  | |  |  |
| **Control** | 98.57±0.733 | 0.21±0.170 | 0.72±0.368 | | 0.51±0.386 | 1.44±0.734 |
| **Doxazosin** |  |  |  | |  |  |
| **75 µM** | 97.23±1.279 | 0.45±0.294 | 1.87±0.915 | | 0.45±0.272 | 2.77±1.28 |
| **180 µM** | 89.09±1.233^**^ | 1.99±1.790 | 6.95±2.121 | | 1.98±1.080 | 10.92±1.232 |
| **250 µM** | 81.73±1.346^***^ | 3.46±1.725 | 9.51±2.510 | | 5.29±0.509 | 18.27±1.346 |
| **Lapatinib** |  |  |  | |  |  |
| **500 nM** | 73.15±1.889^***,##^ | 1.90±0.975 | 17.28±6.134^*^ | | 7.68±3.477 | 26.86±1.885 |

Data are represented as percentage of Means±Standard Error of Means; *p<0.05; **p<0.01; ***p<0.001 in relation to control; ##p<0.01 in relation to 250 µM doxazosin; at least 4 independent experiments; Ann: Annexin V; PI: Propidium Iodide.

|  |  |  |  |  |
| --- | --- | --- | --- | --- |
