# Supplementary material for: Phosphatidylinositol 3-Kinase/AKT Pathway Inhibition by Doxazosin Promotes Glioblastoma Cells Death, Upregulation of p53 and Triggers Low Neurotoxicity
Source: PLoS One. 2016 Apr 28;11(4):e0154612. doi: 10.1371/journal.pone.0154612 (PMC4849739; doi:10.1371/journal.pone.0154612)
Supplement: S2 Table — (DOC) [file pone.0154612.s005.doc]

**Supplementary Table 2** Descriptive statistics of percentage of cell death on glioma cell lines.

|  | | **AnV-/PI-** | | **AnV+/PI-** | | **AnV-/PI+** | | | **AnV+/PI+** | | **Total Cell Death** |
| --- | --- | --- | --- | --- | --- | --- | --- | --- | --- | --- | --- |
| **C6** | | | | | | |  | | | | |
|  |  | |  | |  | | |  | |  | |
| **Control** | 96.85±0.437 | | 0.86±0.130 | | 1.68±0.226 | | | 0.62±0.091 | | 3.16±0.435 | |
| **Vehicle** | 93.90±1.987 | | 3.22±1.804 | | 1.59±0.244 | | | 1.29±0.370 | | 6.11±1.985 | |
| **Doxazosin** |  | |  | |  | | |  | |  | |
| **50 µM** | 85.97±3.825 | | 8.49±2.689 | | 2.46±0.207 | | | 3.08±0.959 | | 14.03±3.825 | |
| **100 µM** | 63.32±5.047^***^ | | 9.11±1.784 | | 17.24±0.630^*^ | | | 10.33±3.924 | | 36.68±5.045 | |
| **150 µM** | 47.43±2.991^***^ | | 14.28±3.556^**^ | | 13.92±2.680 | | | 24.38±2.121^***^ | | 52.57±2.998 | |
| **180 µM** | 35.29±3.440^***^ | | 14.89±1.581^**^ | | 28.62±6.373^***^ | | | 26.20±4.860^***^ | | 69.71±2.503 | |
| **Lapatinib** |  | |  | |  | | |  | |  | |
| **500 nM** | 52.04±0.973^***,#^ | | 5.39±2.167^#^ | | 20.56±1.591^**^ | | | 22.01±1.548^***^ | | 47.96±0.975 | |
|  |  | |  | |  | | |  | |  | |
| **U138-MG** | | | | | | |  | | | | |
|  | |  | |  | |  | | |  | |  |
| **Control** | | 95.65±1.476 | | 1.69±0.671 | | 0.88±0.118 | | | 1.78±0.701 | | 4.35±1.471 |
| **Vehicle** | | 95.46±1.735 | | 1.40±0.560 | | 1.00±0.024 | | | 2.14±1.191 | | 4.54±1.739 |
| **Doxazosin** | |  | |  | |  | | |  | |  |
| **15 µM** | | 87.44±0.560 | | 2.95±0.471 | | 2.19±0.413 | | | 7.42±0.896 | | 12.56±0.562 |
| **30 µM** | | 83.00±1.431 | | 4.59±2.322 | | 6.45±4.907 | | | 5.96±3.245 | | 17.00±1.431 |
| **50 µM** | | 64.10±3.359^***^ | | 5.20±0.183 | | 2.39±0.069 | | | 28.32±3.144^***^ | | 35.90±3.356 |
| **75 µM** | | 47.31±5.709^***^ | | 6.38±3.041 | | 4.45±3.270 | | | 41.86±5.589^***^ | | 52.69±5.709 |
| **Lapatinib** | |  | |  | |  | | |  | |  |
| **500 nM** | | 58.46±0.399^***^ | | 0.17±0.047 | | 36.13±1.733^***,###^ | | | 5.14±1.573^###^ | | 41.44±0.466 |

Data are represented as percentage of Means±Standard Error of Means; *p<0.05; **p<0.01; ***p<0.001 in relation to control; ##p<0.01 in relation to 75 or 180 µM doxazosin; at least 4 independent experiments; AnV: Annexin V; PI: Propidium Iodide.
